# 840. Risk-Based Screening on Admission Cultures (SOAC) vs. Unit-Wide Reactionary Surveillance Cultures (UWRC) for *Candida auris*: A Pilot QI Study in a Tertiary Care Center ICU

**DOI:** 10.1093/ofid/ofad500.885

**Published:** 2023-11-27

**Authors:** Nora Strong, Bela Patel, LaRhonda Daniels, Valerie Ausborn, Elizabeth Mitchell, Chris Denman, Toni Von Wenckstern, Misti G Ellsworth, Luis Ostrosky-Zeichner, Audrey Wanger

**Affiliations:** University of Texas Health Science Center at Houston, Houston, Texas; University of Texas Health Science Center, Houston, Texas; Memorial Hermann Texas Medical Center, Houston, Texas; Memorial Hermann Health System, Houston, Texas; Memorial Hermann Texas Medical Center, Houston, Texas; Memorial Hermann Texas Medical Center, Houston, Texas; Memorial Hermann Texas Medical Center, Houston, Texas; UTHealth, Houston, Texas; McGovern Medical School. UTHealth, Texas, Texas; McGovern Medical School, Houston, Texas

## Abstract

**Background:**

*Candida auris* (*C. auris*) is a highly pathogenic, often multidrug resistant organism, that spreads in healthcare facilities. CDC recommends UWRC when a case is found in a unit. Currently, no recommendation exists for *C. auris* SOAC. We investigated if UWRC can be avoided, and clinical cases be prevented by SOAC in high-risk patients being admitted to an ICU.

**Methods:**

We conducted a pilot QI study in which high-risk patients for *C. auris* colonization (defined as those admitted from long term care facilities, a history of echinocandin use in the last 3 months, or a history of *C. auris* infection or colonization) were screened for skin colonization with fungal axillary and groin swab cultures. These patients were placed on contact precautions pending test results. If *C. auris* was recovered, the patient would be upgraded to “Contact plus” precautions. If cultures were negative, precautions were downgraded to standard. No UWRC would be required if patients were identified and isolated upfront. We tracked the number of SOAC and clinical cases and compared them to the number of UWRC and clinical cases in the pre-study period.

**Results:**

Between January 2023 and March 2023, an average of 273 *C. auris* UWRCs were performed per month in reaction to 18 clinical cases, with 8 positive surveillance cultures over those 3 months (0.98% positive rate). In April 2023, our pilot study month, a total of 13 admission screening cultures were collected, of which none were positive. No UWRC were performed, and no clinical cases were detected.

**Conclusion:**

Although limited by the pre- and post-nature, the abbreviated time, and the sample size of this pilot QI study, we found that risk based SOAC is a strategy that warrants further study as an alternative to UWRC, which are costly and labor intensive.

**Disclosures:**

**Luis Ostrosky-Zeichner, MD, FACP, FIDSA, FSHEA, FECMM, CMQ**, Astellas: Grant/Research Support|Cidara: Advisor/Consultant|Cidara: Advisor/Consultant|F2G: Advisor/Consultant|Gilead: Advisor/Consultant|Gilead: Grant/Research Support|GSK: Advisor/Consultant|Melinta: Advisor/Consultant|NIH: Grant/Research Support|Pfizer: Advisor/Consultant|Pfizer: Grant/Research Support|Pfizer: Honoraria|Pulmocide: Grant/Research Support|Scynexis: Grant/Research Support|T2 Biosystems: Grant/Research Support|Viracor: Advisor/Consultant

